# Characterization of cerebrospinal fluid and peripheral blood immune niche in a patient with primary neurolymphomatosis

**DOI:** 10.1016/j.gendis.2026.102198

**Published:** 2026-04-21

**Authors:** Xinyang Li, Qiguo Zhang, Jing Guo, Jingjing Wang, Ying Xie, Huanhuan Liu, Wenjing Li, Hang Chen, Yuzhen Cui, Mingru Jiang, Christina He, Zhiqiang Liu, Wenrong Huang

**Affiliations:** a Shandong Provincial Key Laboratory of Precision Oncology, Shandong Cancer Hospital and Institute, Shandong First Medical University and Shandong Academy of Medical Sciences, Jinan, Shandong 250117, China; b Department of Hematology, The First People’s Hospital of Chuzhou, Chuzhou Hospital Affiliated to Anhui Medical University, Chuzhou, Anhui 239000, China; c Department of Hematology, Nanjing Drum Tower Hospital, Nanjing University, Nanjing, Jiangsu 210008, China; d Queen Mary School, Nanchang University, Nanchang, Jiangxi 330038, China; e Queen Mary University of London, London E1 4NS, United Kingdom; f Department of Oncology, The First Affiliated Hospital of Dalian Medical University, Dalian Medical University, Dalian, Liaoning 116011, China; g Carnegie Vanguard High School, Houston, TX 77019, USA; h Department of Hematology, Fifth Medical Center of Chinese PLA General Hospital, Beijing 100071, China

Primary neurolymphomatosis (PNL) is a rare hematologic malignancy characterized by the direct infiltration of lymphoma cells into the peripheral nervous system (including nerve roots, peripheral nerves, or cranial nerves), without systemic manifestations of lymphoma.^1^^,^^2^ Its clinical heterogeneity and nonspecific presentations pose significant diagnostic challenges, often leading to misdiagnosis and delayed treatment.^1^ While advances in imaging and molecular techniques have improved diagnostic accuracy,^3^ PNL remains poorly understood at the biological level. Due to its rarity, existing literature is limited to case reports and reviews,^4^ with a critical knowledge gap regarding the immune microenvironment and tumor biology underlying PNL pathogenesis—including how immune cell interactions, clonal dynamics, and metabolic crosstalk drive disease progression and neurotropism. Here, we report a comprehensive single-cell multi-omics analysis of cerebrospinal fluid (CSF) and peripheral blood from a diffuse large B-cell lymphoma (DLBCL)-subtype PNL patient, aiming to fill this gap and generate hypotheses for future research.

A 67-year-old male patient presented with diplopia, multiple cranial nerve palsies, and multiple radiculopathies, alongside rapid disease progression. A moderate number of abnormal cells was observed; the cells were medium-sized with a round to slightly irregular cell body (Fig. S1A). Flow cytometric analysis of the CSF identified a monoclonal B-cell population (CD20^+^, κ-restricted, IGK+) and a markedly elevated IL-10 level of 815 pg/mL. Positron emission tomography-computed tomography (PET-CT) showed increased metabolic activity in multiple nerve roots (Fig. S1B–S1E). No bone marrow involvement was detected. Based on the clinical presentation, imaging, cytologic findings, and molecular evidence, the diagnosis is PNL consistent with the DLBCL subtype. The patient underwent R-MO regimen chemotherapy (Rituximab 700 mg on Day 1; Methotrexate 6.7 g on Day 2; Orelabrutinib 150 mg once daily). After two cycles of treatment, neurological symptoms significantly improved; dysphagia resolved, and facial numbness and limb pain were alleviated. PET-CT and Magnetic Resonance Imaging (MRI) showed complete remission (Fig. 1A). The patient continued subsequent treatment, with follow-up examinations confirming sustained complete remission (Fig. 1B).

**Figure 1:**
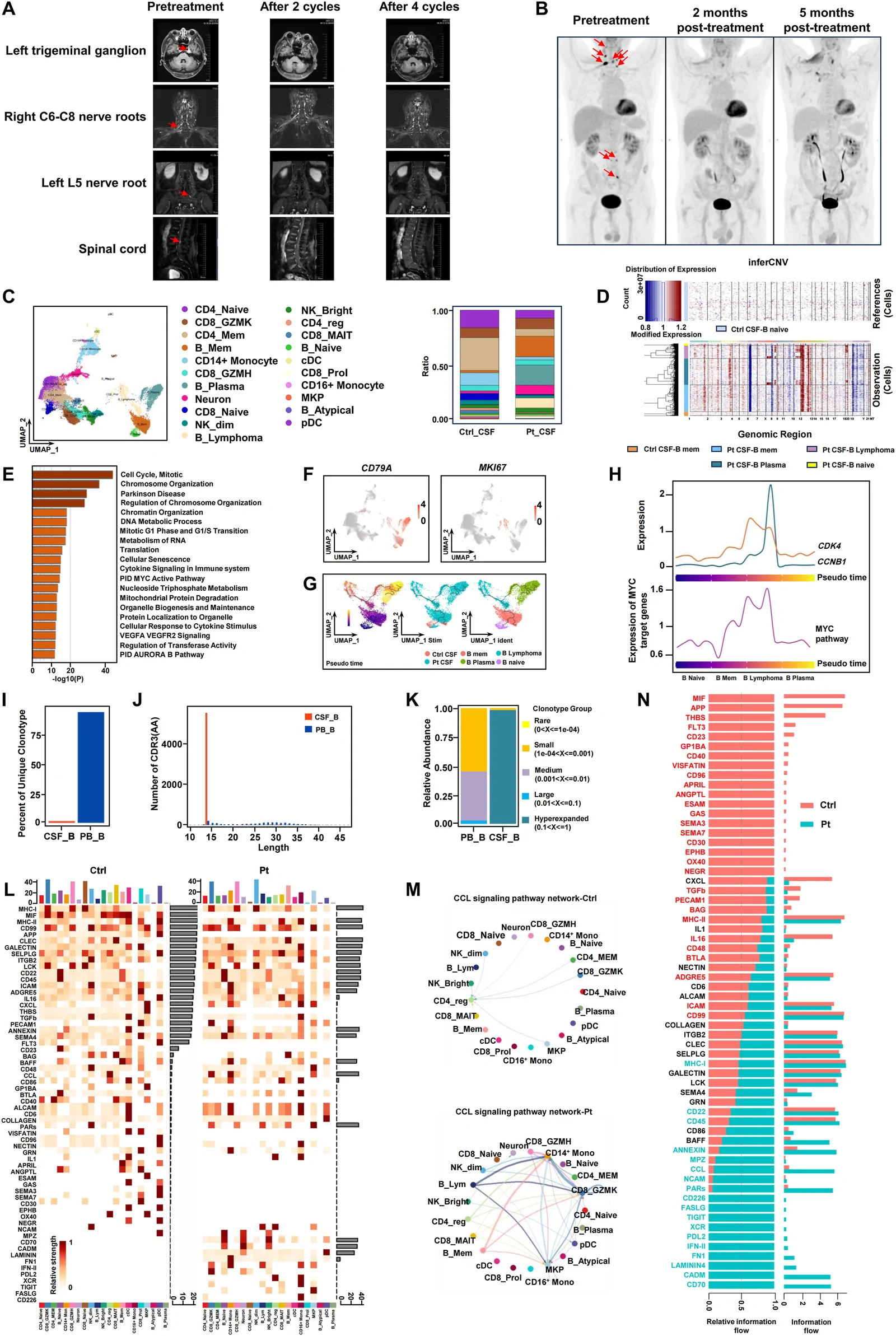
Single-cell RNA-seq assessment of the immune landscape and treatment response in PNL. **(A)** MRI images of the patient’s left trigeminal ganglion, right C6–C8 nerve roots, left L5 nerve root, and spinal cord before treatment, after two cycles of treatment, and after four cycles of treatment. **(B)** PET-CT images of the patient before treatment, two months post-treatment, and four months post-treatment. **(C)** UMAP visualization of immune cell subpopulations in CSF, including naive and memory B cells (B_Naive, B_Mem, B_Plasma), T cell subsets (CD4_Naive, CD4_Mem, CD4_reg, CD8_Prol, CD8_GZMK, CD8_MAIT), monocytes (CD14^+^ Monocyte, CD16^+^ Monocyte), natural killer cells (NK_Bright, NK_dim), dendritic cells (cDC, pDC), neurons, and B_Lymphoma cells. Colors represent distinct cell clusters. Bar plot showing the relative abundance of immune cell subpopulations in Ctrl_CSF and Pt_CSF. The *y*-axis indicates the ratio of each cell type in the total population. **(D)** Heatmap of CNV profiles for individual cells from B cell subsets (B_Naive, B_Mem, B_Plasma) in Ctrl_CSF and Pt_CSF. Red: chromosome amplifications; Blue: chromosome deletions. **(E)** GO pathway enrichment analysis of differentially regulated genes in Pt_CSF compared to Ctrl_CSF. The *y*-axis lists enriched pathways (e.g., Cell Cycle, Chromatin Organization, Cytokine Signaling in Immune System), and the *x*-axis shows –log10 (*P*) values, indicating enrichment significance. **(F)** Visualization of the enriched regions of the *CD79A* and *MKI67* genes. **(G, H)** Pseudotime trajectory analysis of B cell subsets (B_Naive → B_Mem → B_Plasma → B_Lymphoma) in CSF, showing the expression dynamics of cell cycle-related genes (*CDK4*, *CCNB1*) and MYC pathway target genes along differentiation. **(I)** Bar plot comparing the percentage of unique clonotypes in CSF B cells (CSF_B) and peripheral blood B cells (PB_B). **(J)** Distribution of CDR3 amino acid (AA) length in CSF_B and PB_B, with the *x*-axis indicating length and the *y*-axis showing the number of clonotypes. **(K)** Relative abundance of clonotype groups (Rare, Small, Medium, Large, Hyperexpanded) in CSF_B and PB_B, defined by clonotype frequency ranges. **(L)** Heatmaps show the relative strength of overall signaling patterns (color intensity: red = high; white = low) in control (Ctrl, left) and patient (Pt, right) cerebrospinal fluid (CSF). Key interactions (e.g., adhesion molecules, cytokine signaling) differ between groups: compared with Ctrl, the overall signaling pattern in Pt shifted from CXCL/IL16 to CCL, CD70, CADM, and LAMININ, with altered interaction strength in patient-derived B lymphoma cells and associated immune subsets. **(M)** Cell–cell signaling pathway network of CCL in Ctrl (left) and Pt (right) samples, depicting altered interaction patterns. **(N)** Relative information flow of critical signaling pathways (e.g., COLLAGEN, ALCAM, ICAM) in Ctrl *vs.* Pt CSF, showing differential activation. MRI: Magnetic Resonance Imaging, PET-CT: Positron Emission Tomography-Computed Tomography, UMAP: Uniform Manifold Approximation and Projection, NK: natural killer cells, DC: dendritic cells, CSF: cerebrospinal fluid, CNV: copy number variation, GO: Gene Ontology, CDR3: complementarity determining region 3, CCL: chemoattractant cytokine ligand, CSF: cerebrospinal fluid.

We performed single-cell RNA sequencing on CSF and peripheral blood from the patient, using GSE243905 as a healthy control, and integrated TCR and BCR sequencing. Compared with controls, the patient’s CSF showed decreased proportions of CD4^+^ T cells, CD8^+^ T cells, and CD14^+^ monocytes, while B cell subsets (including a B-lymphoma cells cluster, memory B cells, and plasma cells) were markedly increased (Fig. 1C; Fig. S2A and S2B). Copy number variation (CNV) analysis revealed high genomic instability in the CSF B cells (Fig. 1D). Gene Ontology analysis showed that patient-specific B cell genes were enriched for cell cycle, chromosome organization, and proliferation pathways, with *MKI67* predominantly expressed in the B-lymphoma subset (Fig. 1E and F). Pseudotime trajectory analysis suggested that B-lymphoma cells underwent an evolutionary process involving naive B cell subsets and memory B cell subsets, with a portion of B-lymphoma cells differentiating toward the B-plasma subset (Fig. 1G). Furthermore, it also demonstrated dynamic upregulation of cell-cycle regulators (e.g., *CCNB1*, *CDK4*) and MYC target genes in the B-lymphoma subgroup, consistent with rapid proliferation (Fig. 1G and H). BCR clonotype analysis indicated dominant clonal expansion of CSF B cells, a single CDR3 length peak at 14 amino acids, and significantly reduced Shannon and Simpson diversity indices, reflecting high clonality (Fig. 1I–K). Patient peripheral blood also showed significant CNVs in B cells and an increased proportion of CD14^+^ monocytes, whose specific genes were enriched in cytokine-signaling pathways (e.g., interferon-gamma and interleukin-12 family signaling) (Fig. S2C, S2D, S3A–S3D).

A pronounced proliferative CD8^+^ T cell subset was present in the patient’s CSF, expressing proliferation markers (*MKI67*) and enriched for cell-cycle pathways (Fig. S3E and S3F). TCR analysis showed reduced overall clonal diversity in CSF T cells (lower Shannon/Simpson indices), while the CDR3 length distribution remained similar to that of controls, indicating that the proliferative CD8^+^ subset is reactive rather than highly clonal (Fig. S3G–S3I).

Analysis of intercellular communication networks revealed an altered pattern of signaling, with information flow in the patient’s CSF concentrating in adhesion-related pathways (NCAM, CADM, LAMININ) (Fig. 1L). Interactions among the proliferative CD8^+^ subset, B-lymphoma cells, and CD14^+^ monocytes were markedly strengthened in the tumor niche (Fig. S3J), with increased pro-inflammatory and migration-related signaling (e.g., CD226, IFN II, CXCL, CCL, GAL-LECTIN, XCR) and the presence of immunosuppressive signals (e.g., TIGIT) (Fig. 1L–N). CCL signaling networks showed frequent interactions among CD14^+^ monocytes, B-lymphoma, memory B, and CD8^+^ GZMK cells (Fig. 1M), and upregulated adhesion-related ligand–receptor pairs (e.g., LGALS9-CD44/CD45, ITGB2-CD226, LGALS9-HAVCR2) linking CD14^+^ monocytes, B lymphoma, and proliferative CD8^+^ cells (Fig. S3K). Despite stronger specific interactions, the overall cell–cell communication diversity was reduced (Fig. S3L), suggesting that B cell clonal expansion may impair the breadth of intercellular signaling.

Metabolic communication analysis suggested CD14^+^ monocytes as the principal metabolite donors (most sending events and many receiving events), with B-lymphoma and proliferative CD8^+^ subsets also highly involved (Fig. S3M–S3O). CD14^+^ monocytes were predicted to primarily supply l-glutamine to B-lymphoma cells via the expression of amino acid transporters (SLC3A2, SLC38A1, SLC38A2), with a lesser contribution from proliferative CD8^+^ cells inferred from scRNA-seq data—although direct metabolite flux was not experimentally confirmed (Fig. S3P). CD14^+^ monocytes had high levels of choline, ADP-ribose, and cholesterol, while B-lymphoma and proliferative CD8^+^ subsets showed elevated ADP levels (Fig. S3Q). B-lymphoma cells obtained amino acids, d-fructose, and choline from multiple microenvironmental sources via amino-acid transporters and acquired iron through the transferrin receptor to support survival and proliferation (Fig. S3R).

From the perspective of single-cell multi-omics analysis, this single-case study may uncover key molecular features of PNL, including prominent B cell clonality, enhanced adhesion signaling networks, and metabolic dependencies (predicted l-glutamine supply from CD14^+^ monocytes to B-lymphoma cells based on amino acid transporter expression). These insights may predict potential mechanisms underlying PNL neurotropism—adhesion molecules may facilitate lymphoma cell infiltration into peripheral nerves, while metabolic crosstalk is predicted to support tumor survival in the nervous system niche. Additionally, tumor microenvironment metabolism, targeted adhesion/migration molecules, and combined immune checkpoint inhibitors might be promising for future diagnostic biomarker development (direct utility not investigated). The patient’s sustained complete remission with the R-MO regimen provides preliminary support for targeting malignant B cells and their supportive microenvironment, but generalizability requires validation in larger cohorts. This study has several important limitations that must be acknowledged. First, the findings are based on a single patient with DLBCL-subtype PNL. PNL is a highly heterogeneous disease, with variations in lymphoma subtype, anatomical involvement, clinical course, and genetic background across patients.^5^ As such, the immune signatures, genomic features, and intercellular communication patterns identified here may not be generalizable to all PNL cases. The single-case design precludes statistical analysis of the prevalence or clinical relevance of these features, and we cannot rule out that some observations are patient-specific rather than disease specific. Additionally, all our results are speculative, derived solely from computational and correlative analyses of sequencing data, and lack validation through *in vitro* functional assays or *in vivo* experimental models.

In summary, single-cell multi-omics analysis reveals distinct immune landscape alterations, clonal B-cell expansion, enhanced adhesion/pro-inflammatory signaling, and metabolic crosstalk in the CSF of PNL patients. These findings provide insights into PNL pathogenesis and potential therapeutic targets.

## CRediT authorship contribution statement

**Xinyang Li:** Writing – original draft, Formal analysis. **Qiguo Zhang:** Writing – original draft, Conceptualization. **Jing Guo:** Writing – original draft, Funding acquisition. **Jingjing Wang:** Investigation, Funding acquisition. **Ying Xie:** Investigation, Funding acquisition. **Huanhuan Liu:** Methodology, Investigation. **Wenjing Li:** Data curation. **Hang Chen:** Data curation. **Yuzhen Cui:** Data curation. **Mingru Jiang:** Data curation. **Christina He:** Data curation. **Zhiqiang Liu:** Writing – review & editing, Supervision, Project administration, Methodology, Funding acquisition, Formal analysis, Conceptualization. **Wenrong Huang:** Writing – review & editing, Project administration, Conceptualization.

## Ethics declaration

This study was approved by the Ethics Committee of Shandong Cancer Hospital and Research Institute (SDTHEC-2024001050), and all protocols conformed to the Ethical Guidelines of the World Medical Association Declaration of Helsinki. Signed informed consent was obtained from each participating individual prior to participation in the study. The patient signed the informed consent form, and the study was approved by the Ethics Committee.

## Data availability

The RNA-seq data can be found publicly in the Gene Expression Omnibus database under accession number GSE243905. All data are available in the main text or the Supplementary materials, and are available from the corresponding author upon reasonable request.

## Funding

This work was supported by the 10.13039/501100001809National Natural Science Foundation of China (No. 82500275 to JG, 82300229 to YX, 82370209, 82570254 to ZQL, 82400243 to JJW), the Taishan Scholar Program of Shandong Province, China (No. tstp20230632 to ZQL), the Anhui University Natural Science Research Project (No. 2023AH040094 to QGZ), and the Collaborative Academic Innovation Project of Shandong Cancer Hospital (China) (No. FC009 to ZQL).

## Conflict of interests

The authors declare no conflict of interests.
