# Supplementary material for: Characterization of cerebrospinal fluid and peripheral blood immune niche in a patient with primary neurolymphomatosis
Source: Genes Dis. 2026 Apr 21;14(3):102198. doi: 10.1016/j.gendis.2026.102198 (PMC13740842; doi:10.1016/j.gendis.2026.102198)
Supplement: Supplementary file 1 [file mmc1.docx]

**Supplementary methods**

**Patients and Ethic Approval**

A 67-year-old male patient presented to the Fifth Medical Center of Chinese PLA General Hospital in 2023. Flow cytometry of his cerebrospinal fluid revealed abnormal B lymphocytes, and PET-CT indicated areas of increased neuro-metabolic activity. Single-cell sequencing, TCR sequencing, and BCR sequencing were performed on samples from blood and cerebrospinal fluid. Data from the healthy control group in the Gene Expression Omnibus database (GSE189460) were used as the control group for this study. This study was approved by the Ethics Committee of Shandong Cancer Hospital and Research Institute (SDTHEC-2024001050), and all protocols conformed to the Ethical Guidelines of the World Medical Association Declaration of Helsinki. Signed informed consent was obtained from each participating individual prior to participation in the study.

**Analysis of single-cell RNA sequencing data**

Raw data from sequencing were processed using an internal pipeline to generate gene expression matrixes. We excluded low-quality cells by removing those with fewer than 100 unique genes, fewer than 500 unique molecular identifiers (UMI) reads, and more than 30% mitochondrial UMIs. FastQC v0.11.4 and fastp84 were applied to process raw reads and remove doublets (cells with double amounts of unique molecular identifiers (UMIs)) by keeping only cells with <10,000 UMI reads, and filter out cells with high percentages of mitochondrial genes (>10%). Cutadapt85 was used to trim poly-A tail and adapter sequences. And then Cell barcode and UMI were extracted. Later, Reads were mapped to the reference genome GRCh38 (ensembl version 92 annotation) by STAR86 v2.5.3a. UMI counts and gene counts per cell were obtained using featureCounts87 v1.6.2 to generate expression matrix. Analysis was performed in R (v4.1.3) using the package Seurat (v4.1.1). QC was performed in Seurat. To mitigate batch effects arising from potential technical differences (e.g., sequencing run, sample handling) between the patient dataset and the public control dataset GSE243905, we applied the Harmony algorithm (v0.10.0) for data integration. The package ggplot2 (v3.3.6) was used to generate figures. Doublets were removed by DoubletFinder (v2.0.3) and singlets were used for downstream quality control. Cells with high percentages of mitochondrial genes (>10%) were filtered out. The cells in the resulting integrated assay were visualized onto a two-dimensional map to reveal heterogeneity among cells using a Uniform Manifold Approximation and Projection (UMAP) embedding. Similar cells were clustered together via Louvain clustering on the top principal components of the integrated assay using Seurat package.

Differentially expressed genes (DEGs) in each cluster were identified by the Seurat function FindMarkers, filtered using a minimum fold change of 1.5 and a maximum adjusted p value of 0.05 and were then ranked by average log2(fold change) and FDR. Results were visualized by volcano plot. The Metascape webtool (www.metascape.org). was used to accomplish the GO analysis of DEGs, P values of GO terms and gene ratio were calculated based on the accumulative hypergeometric distribution by the Metascape webtool.

**Cellular communication and interaction analysis**

To understand how B lymphoma cells interact with each other, we used CellChat software platform to infer the interactions. We next ranked the ligand expressed on the target cells by the gene expression proportion and frequency. Information flow characterizes the possibility of cell–cell interaction mediating through a known pathway. To compare the overall signaling structure between cells in control and patient samples, interaction weights were used, which sum the information flow of all Ligand-Recptor (L-R) interactions between two cell types. To compare the signaling structure of individual L-R interactions, we first used CellChat network embedding feature to identify pathways with large differences in the sources or targets between control and patient groups. We used a functional embedding, which creates a two-dimensional embedding of all L-R interactions based on the gene expression of the source and target cells involved in an individual L-R network. The Euclidean distance between the same L-R pathway in groups was used to identify pathways with large differences in signaling structure.

**Cell trajectory analysis**

We used monocle3 to infer the pseudotime and plot the differentiation state of B cells. Trajectories were estimated using the standard Monocle 3 workflow. Trajectories were learned using the default parameter ‘learn_graph’ function. B_Naive subset in the trajectory with the earliest time point in the vicinity was assigned as root node.
