# Supplementary material for: Characterization of cerebrospinal fluid and peripheral blood immune niche in a patient with primary neurolymphomatosis
Source: Genes Dis. 2026 Apr 21;14(3):102198. doi: 10.1016/j.gendis.2026.102198 (PMC13740842; doi:10.1016/j.gendis.2026.102198)
Supplement: Supplementary file 2 [file mmc2.docx]

**Figure legends**

**Figure S1. Multimodal imaging analysis of a patient**

**(A)** Histopathological images of cerebrospinal fluid (CFS) smears, showing cellular morphology under a light microscope.

**(B)** Top left: Coronal view showing whole - body metabolic distribution, with physiological tracer uptake in organs like the heart and bladder. Top right: Axial view highlighting focal tracer accumulation of the right C6 nerve root. Bottom left: CT image providing anatomical details of bony structures, soft tissues, and air - filled spaces. Bottom right: Fused PET - CT image, where the color overlay (e.g., red - hotted hotspots) maps metabolic activity (from PET) onto anatomical context (from CT), highlighting focal tracer accumulation of the right C6 nerve root.

**(C)** Top left: Coronal view showing whole - body metabolic distribution, with physiological tracer uptake in organs like the heart and bladder. Top right: Axial view highlighting focal tracer accumulation of the right T1 nerve root. Bottom left: CT image providing anatomical details of bony structures, soft tissues, and air - filled spaces. Bottom right: Fused PET - CT image, where the color overlay (e.g., red - hotted hotspots) maps metabolic activity (from PET) onto anatomical context (from CT), highlighting focal tracer accumulation of the right T1 nerve root.

**(D)** Top left: Coronal view showing whole - body metabolic distribution, with physiological tracer uptake in organs like the heart and bladder. Top right: Axial view highlighting focal tracer accumulation of the left L3 nerve root. Bottom left: CT image providing anatomical details of bony structures, soft tissues, and air - filled spaces. Bottom right: Fused PET - CT image, where the color overlay (e.g., red - hotted hotspots) maps metabolic activity (from PET) onto anatomical context (from CT), highlighting focal tracer accumulation of the left L3 nerve root.

**(E)** Top left: Coronal view showing whole - body metabolic distribution, with physiological tracer uptake in organs like the heart and bladder. Top right: Axial view highlighting focal tracer accumulation of the left L5 nerve root. Bottom left: CT image providing anatomical details of bony structures, soft tissues, and air - filled spaces. Bottom right: Fused PET - CT image, where the color overlay (e.g., red - hotted hotspots) maps metabolic activity (from PET) onto anatomical context (from CT), highlighting focal tracer accumulation of the left L5 nerve root.

CFS: cerebrospinal fluid, PET: Positron Emission Tomography, CT: Computed Tomography, PET-CT: Positron Emission Tomography-Computed Tomography.

**Figure S2.** **Immune landscape in peripheral blood and CSF**

**(A)** UMAP visualization of immune cell distribution in Ctrl_CSF and Pt_CSF, showing subsets including B cells, T cells, monocytes, and NK cells. Relative abundance of immune cell subpopulations in Ctrl_CSF vs. Pt_CSF, with ratios indicating shifts in cell frequencies.

**(B)** Dot plot showing the expression of marker genes across immune cell subsets in CSF. Dot color represents average expression level (gradient from purple to yellow), and dot size indicates the percentage of cells expressing the gene within each subset, grouped by cell lineages.

**(C)** UMAP visualization of immune cell distribution in Ctrl_Blood and Pt_Blood, showing subsets including B cells, T cells, monocytes, and NK cells. Relative abundance of immune cell subpopulations in Ctrl_Blood vs. Pt_Blood, with ratios indicating shifts in cell frequencies.

**(D)** Dot plot showing the expression of marker genes across immune cell subsets in peripheral blood. Dot color represents average expression level (gradient from purple to yellow), and dot size indicates the percentage of cells expressing the gene within each subset, grouped by cell lineages.

UMAP: Uniform Manifold Approximation and Projection, NK: natural killer cells, CSF: cerebrospinal fluid.

**Figure S3.** **Immune profiling across CSF and peripheral blood**

**(A)** Shannon and Inverse Simpson indices for B cell clonotype diversity in CSF_B and PB_B.

**(B)** Heatmap of CNV profiles for individual cells from B cell subsets (B_Naive, B_Mem, B_Plasma) in Ctrl Blood and Pt Blood. Red: chromosome amplifications; Blue: chromosome deletions.

**(C)** Stacked bar plot comparing the ratio of various immune cell subsets in Ctrl Blood (blue) and Pt Blood (red).

**(D)** Bar plot of GO and pathway enrichment results of CD14^+^ monocytes in peripheral blood. The y - axis lists biological processes/pathways (e.g., Cytokine Signaling in Immune System, Neutrophil Degranulation), and the x - axis shows - log10(P) values, indicating enrichment significance by bar length and color gradient.

**(E)** Visualization of the enriched regions of *CD8A* and *MKI67* genes.

**(F)** GO pathway enrichment analysis focusing on T cell-related biological processes in CSF T cells (CSF_T), including Cell Cycle, DNA Repair, and Cellular Senescence. The x-axis shows -log10(P) values for enrichment significance.

**(G)** CDR3 AA length distribution of T cell receptors (TRA, TRB) in Ctrl CSF_T (n=3) and Pt CSF_T.

**(H)** Relative abundance of T cell clonotype groups in Ctrl CSF_T and Pt CSF_T, categorized by frequency.

**(I)** Shannon and Inverse Simpson indices for T cell clonotype diversity in Ctrl CSF_T and Pt CSF_T.

**(J)** Scatter plots comparing incoming vs. outgoing interaction strengths of immune cell subsets in Ctrl and Pt groups. Points represent cell types (e.g., CD14^+^ Monocyte, B_Lymphoma), with size indicating cell count and color distinguishing subsets.

**(K)** Dot plot showing ligand - receptor interactions between immune cell subsets in Pt samples. Rows represent ligand - receptor pairs, columns represent sender - receiver cell combinations. Dot color indicates communication probability (gradient from min to max), and size reflects p - value significance (small: 0.01 < p < 0.05; large: p < 0.01).

**(L)** Total number of inferred interactions and cumulative interaction strength in Ctrl_CSF vs. Pt_CSF, showing decreased immune crosstalk in patients (3745 interactions, strength=196.031 in Ctrl vs. 3395 interactions, strength=167.614 in Pt).

**(M)** The number of communications for each cell group, with purple bars representing sender events and orange bars representing receiver events, across different cell group identifiers on the x - axis.

**(N)** A cell - cell communication network among various immune cell subsets. Nodes represent cell types (e.g., CD8_MAIT, B_Mem), node colors correspond to overall communication scores (gradient from blue to red), node sizes reflect the number of connected nodes, and edge thicknesses indicate the number of communication events.

**(O)** Heatmap illustrating cell - cell communication between senders (x - axis) and receivers (y - axis). Dot color represents overall communication score (blue - low, red - high), dot size indicates number of communication events (small - 1, medium - 2, large - 10), and dendrograms show hierarchical clustering of senders and receivers.

**(P)** Dot plot of metabolite - transporter/receptor communication pairs. Each dot represents a sender - receiver cell group pair. Dot color indicates - log10(p - value) (blue - low, red - high), size reflects communication score, and x - axis lists sender - receiver cell group indices for pairs like L-Serine - SLC3A2, L-Glutamine - SLC3A2 etc.

**(Q)** Violin plot showing the mean abundance distribution of metabolites (Choline, ADP, Adenosine diphosphate ribose, D - Fructose, Cholesterol) across cell groups (x - axis). The color intensity of the ridgelines represents the mean abundance, with the scale on the right.

**(R)** Network diagram of metabolite - mediated cell communication. Nodes represent senders, metabolites, sensors (transporters/receptors), and receivers. Edges show communication paths, with edge color indicating -log10(p - value), edge width reflecting communication score, and node size denoting connection count.

CNV: copy number variation, GO: Gene Ontology, CSF: cerebrospinal fluid, DNA: Deoxyribonucleic Acid, CDR3: Complementarity Determining Region 3, AA: amino acid, ADP: adenosine diphosphate.
